# Is Neuronal Histamine Signaling Involved in Cancer Cachexia? Implications and Perspectives

**DOI:** 10.3389/fonc.2019.01409

**Published:** 2019-12-20

**Authors:** Hannes Zwickl, Elisabeth Zwickl-Traxler, Martin Pecherstorfer

**Affiliations:** Department of Internal Medicine 2, University Hospital Krems, Karl Landsteiner Private University of Health Sciences, Krems, Austria

**Keywords:** cancer cachexia, central histaminergic system, histaminergic neurons, histamine signaling, food intake, energy expenditure, parasympathetic nervous system, anorexia

## Abstract

In this paper, we present evidence in support of our hypothesis that the neuronal histaminergic system might be involved in cancer cachexia^1^. To build our premise, we present the research and the reasonable inferences that can be drawn from it in a section by section approach starting from one of the key issues related to cachexia, increased resting energy expenditure (REE), and progressing to the other, anorexia. Based on an extensive survey of the literature and our own deliberations on the abovementioned topics, we investigate whether histamine signaling might be the mechanism used by a tumor to hijack the body's thermogenic machinery. Our hypothesis in short is that hypothalamic histaminergic neurons are stimulated by inputs from the parasympathetic nervous system (PSNS), which senses tumor traits early in cancer development. Histamine release in the preoptic area of the hypothalamus primarily activates brown adipose tissue (BAT), triggering a highly energy demanding mechanism. Chronic activation of BAT, which, in this context, refers to intermittent and/or low grade activation by the sympathetic nervous system, leads to browning of white adipose tissue and further enhances thermogenic potential. Aberrant histamine signaling not only triggers energy-consuming processes, but also anorexia. Moreover, since functions such as taste, smell, and sleep are governed by discrete structures of the brain, which are targeted by distinct histaminergic neuron populations even relatively minor symptoms of cachexia, such as sleep disturbances and taste and smell distortions, also might be ascribed to aberrant histamine signaling. In late stage cachexia, the sympathetic tone in skeletal muscle breaks down, which we hypothesize might be caused by a reduction in histamine signaling or by the interference of other cachexia related mechanisms. Histamine signaling thus might delineate distinct stages of cachexia progression, with the early phase marked by a PSNS-mediated increase in histamine signaling, increased sympathetic tone and symptomatic adipose tissue depletion, and the late phase characterized by reduced histamine signaling, decreased sympathetic tone and symptomatic muscle wasting. To support our hypothesis, we review the literature from across disciplines and highlight the many commonalities between the mechanisms underlying cancer cachexia and current research findings on the regulation of energy homeostasis (particularly as it relates to hypothalamic histamine signaling). Extrapolating from the current body of knowledge, we develop our hypothetical framework (based on experimentally falsifiable assumptions) about the role of a distinct neuron population in the pathophysiology of cancer cachexia. Our hope is that presenting our ideas will spark discussion about the pathophysiology of cachexia, cancer's devastating and intractable syndrome.

## Introduction

Affecting patients' quality of life, prognosis, and response to and tolerance of therapies (1–3), cachexia is a major issue in cancer management, all the more so as effective treatment options to halt or reverse wasting are still lacking. Cancer-associated body weight loss reflects the profound dysregulation of energy homeostasis, which manifests in increased resting energy expenditure (REE) and in anorexia (1, 2, 4, 5). Moreover, cancer cachexia is associated with perturbations of lipid, glucose, and protein metabolism, and involves most, if not all, organs (6–8).

Systemic inflammation has been identified as a major driver of cancer cachexia (9–11), with cancer-associated anorexia having been attributed to neuroinflammation resulting from the effects of dysregulated cytokine networks on neurons, microglia, and endothelial cells (12–14). Nevertheless, there is consensus that treatment of cancer cachexia requires a multimodal approach and that controlling inflammation alone is a downstream solution targeting symptoms, which is not sufficient to obtain optimal therapy results (15). This indicates that cancer cachexia involves a pathophysiological mechanism triggered by the tumor (16), but not necessarily mediated by tumor-associated systemic inflammation.

A mechanism that meets this criterion recently has been identified in research on the neuronal control of energy homeostasis^2^. Enhanced histamine signaling ameliorates obesity by concomitantly increasing REE and inducing anorexia, the major characteristics of cancer cachexia (17, 18). The central histaminergic system comprises brain mast cells and histaminergic neurons (19), of which the latter are involved in controlling sleep and wakefulness, biological rhythms, mood, and cognition (2, 19–21), all of which may be disrupted in cancer cachexia (2). Indeed, the degree of overlap between the spectrum of physiological functions responsive to neuronal histamine signaling and the clinical manifestations of cancer cachexia is quite remarkable. It appears that in cachexia, neuronal histamine signaling takes on a more prominent role than its mediating function in normal physiology, disconnecting from the biological context normally shaped by other neurotransmitters to the extent that it subverts the action of upstream neurotransmitters and hormones. To wit, neuronal histamine signaling is presumed to work in parallel and largely independent of factors having been implicated in cancer cachexia so far.

We will begin our exploration by taking a closer look at the mechanisms that may play a role in increased REE.

## Increased REE

Increased REE is the main cause of wasting (22). Several organs contribute to REE, including skeletal and cardiac muscle, liver, and both brown and white adipose tissue (BAT and WAT, respectively) (6, 23).

### Activation of BAT

BAT activation has been found in cancer patients (24–27) and its correlation with weight loss demonstrated in rodent models of cancer cachexia (23, 28, 29). However, the significance of activated BAT for cancer cachexia in humans is still a matter of debate. This is mainly due to the fact that ^18^F-fluorodeoxyglucose positron emission tomography/computer tomography (^18^F-FDG-PET-CT), which is used to detect metabolically active BAT in humans, has yielded discrepant results (26, 27). Further studies are required, with appropriate consideration given to variables affecting BAT activity (27). One such potentially significant, but so far neglected, variable is circadian rhythmicity of BAT activation, which has been found in rodent models of cancer cachexia. If the findings in rodents apply to humans, this would suggest that BAT is activated mainly during the night in human cancer cachexia (23, 30). A recent retrospective study on variables influencing BAT activation based on retrospective re-examinations of ^18^F-FDG-PET-CT images from over 15,000 people demonstrated that its incidence changes with time of day (31). Furthermore, since BAT-mediated thermogenesis is a highly energy-demanding process, a slight but persistent increase in BAT activity is sufficient to induce pronounced weight loss (32). One drawback of ^18^F-FDG-PET-CT is that it is not sensitive enough to reliably detect BAT^3^ (33).

**Hypothesis**: Both the time of day of measurement and the lack of sensitivity of ^18^F-FDG-PET-CT might account for the inconsistency of data on BAT activation in cancer cachexia. Data inconsistency also might reflect peculiarities of the underlying mechanism, i.e., circadian rhythmicity and/or low-grade BAT activation. Given that BAT activation data appears to track with circadian rhythms, long-term continuous monitoring of body temperature is needed employing an appropriate measurement technique.

### Browning of WAT

The emergence of beige adipocytes in WAT is associated with cancer cachexia in animal models and humans (24, 25, 34–36). Like brown adipocytes, beige adipocytes express uncoupling protein 1, which uncouples mitochondrial respiration from ATP synthesis in favor of thermogenesis and thereby increases REE (37). However, the extent of its contribution to REE in this context cannot be assessed by ^18^F-FDG-PET-CT due to its limited sensitivity (see above), thus is prone to being underestimated (26). Several humoral factors, the tumorkine parathyroid hormone-related protein (PTHrP) and the cytokine interleukin 6 (IL-6), recently have been suggested as potential causes of WAT browning. Kir et al. identified tumor-derived PTHrP as an inducer of WAT browning in a rodent cancer model and reported that approximately one third of patients with metastatic non-small-cell lung cancer (NSCLC) or colorectal cancer had detectable serum PTHrP, together with higher REE and lower lean body mass, compared to PTHrP-negative patients (34, 38–40). In addition, Hong et al. showed that increased serum PTHrP correlated with increased probability of weight loss in cancer patients, but that weight loss also occurred in the absence of PTHrP. This suggests that PTHrP, rather than being a causative factor, catalyzes already progressive cancer cachexia (41). IL-6, an immanent factor of cachexia-associated systemic inflammation, promotes browning, increases REE, and drives weight loss in cancer cachexia (35). However, though anti-IL-6 therapy alleviates cachectic symptoms, it is most effective in the treatment of cancer cachexia when used as part of a multimodal approach (42).

**Hypothesis**: These research findings indicate that there might be an underlying pathophysiological mechanism, which current treatments for cachectic cancer patients do not yet effectively target. This suggests that tumorkine-mediated mechanisms and systemic inflammation can be active early on as secondary mechanisms contributing to REE, but gain in significance during tumor progression, amplifying tumor-triggered REE and exacerbating systemic wasting in proportion to the rate of tumorkine production and cytokine levels in individual patients. This is consistent with the heterogeneity of cachexia across cancer patients, which manifests as symptoms that vary in type and severity (2).

### Non-shivering Thermogenesis (NST)

In normal physiology, BAT is an effector organ of non-shivering thermogenesis (NST), which is activated by cold stress to maintain body temperature (43). Chronic cold stress promotes the proliferation of brown adipocytes and browning of WAT (37, 44) in rodents (45–47) and humans (48, 49).

**Hypothesis**: Cancer cachexia sharing characteristic features of the body's response to chronic cold stress suggests that a tumor might act as a chronic stressor, triggering the homeostatic response in a manner similar to cold exposure, the physiological stressor. Could NST serve as a blueprint for adipose tissue-driven energy expenditure in cancer cachexia?

NST primarily activates BAT, inducing WAT browning only if cold stress persists (44, 49). This implies that BAT activation precedes WAT browning in normal physiology or, conversely, that browning should not occur without chronically increased BAT activation. However, as discussed earlier, whereas WAT browning has been shown to occur in cancer cachexia (35), BAT activation is still controversial. In humans, intermittent, moderate cold stress^4^—as opposed to continuous triggering of NST or overly pronounced BAT activation—suffices to significantly increase the thermogenic capacity of WAT due to browning (37, 48, 50), with the increase in thermogenic capacity being a function of both duration and extent of the thermogenic trigger (26). In this view, WAT browning, as well as presumed BAT activation, in cachectic cancer patients mirrors the normal function of NST in response to chronic cold stress and aligns with the abovementioned findings re circadian rhythmicity and low-grade activation of BAT.

### Increased Lipolysis

Chronic activation of BAT requires a supply of energy-rich compounds, either lipids from WAT or glucose from the liver (51). NST so effectively removes glucose and free fatty acids (FFA) from systemic circulation (52, 53), and reduces hyperglycemia and hyperlipidemia (54), that its intentional activation already has been attempted to treat obesity and metabolic syndrome (37, 52). The therapeutic application of a moderate cold exposure regimen has been shown to reduce body weight and increase insulin sensitivity in humans (52).

In normal physiology, the high rate of lipid turnover is due to a dynamic equilibrium of lipogenesis and lipolysis in white adipocytes (55). In cancer cachexia, the loss of adipose tissue reflects the depletion of cellular lipid stores (56) and an increase in lipid turnover primarily mediated by accelerated WAT lipolysis (57). Cancer cachexia has been linked to the increased expression of adipose triglyceride lipase and hormone-sensitive lipase, which, once stimulated, increase the lipolytic capacity of white adipocytes (58), while basal lipolysis remains unaltered or is reduced (58). This suggests that pro-lipolytic stimuli actively drive lipid mobilization in cancer cachexia. For instance, zinc-alpha_2_-glycoprotein, a lipid-mobilizing factor, has been associated with cancer cachexia in rodents and humans (59, 60). Insulin is a potent inhibitor of lipolysis, and resistance to its inhibiting effect contributes to increased lipolysis in cancer cachexia (58).

The inverse correlation of WAT-associated triacylglycerol hydrolase activity, the rate-limiting step of lipolysis, with the body mass index of cachectic cancer patients might reflect an adaptation to chronic activation of NST (61). Moreover, the increased FFA oxidation (62) and alterations at the transcriptome level of WAT observed in cachectic cancer patients indicates an increased capacity by white adipocytes to utilize fatty acids and suggests another potential link to browning and concomitant chronicity (63).

In humans, increased lipolysis is mediated mainly by catecholamines and natriuretic peptides (58, 64). In particular, the sympathetic nervous system (SNS) and related noradrenergic signaling are the most important mechanisms for conveying the homeostatic response to cold stress (65, 66). Increased sympathetic tone is sufficient to induce activation of BAT, browning of WAT, and lipolysis (37, 49). In normal physiology, SNS-associated noradrenergic signaling mediates the body's acute stress response^5^. If this stress response were to become chronic, its normal physiological role would be perverted and the mechanisms it triggers—now chronic—would increase REE and cause the loss of body weight, exactly as happens in cachexia.

**Hypothesis**: Consistent with increased lipolysis, plasma levels of triglycerides, FFA, glycerol, and cholesterol have been reported to increase in cancer patients who are losing weight (7, 56). However, other studies found lower serum cholesterol levels in newly diagnosed cancer patients and lung cancer patients (68, 69) and either the same or lower triglyceride levels in lung cancer patients (69) in comparison to non-malignant cohorts. NST is a highly energy-demanding process, thus serum lipid levels might change inversely with NST activation. The reduction in cholesterol levels of cancer patients after an overnight fast, as observed by us (publication in preparation) and others, is consistent with increased NST during the night and correlates with the circadian rhythmicity of BAT activation (68). As a consequence, analogous to BAT activation (see above), the time of day of measurement might be a crucial determinant of plasma cholesterol level.

It has long since been recognized that cholesterol levels begin to decline years before cancer diagnosis. This finding led to the hypothesis that the change in cholesterol reflects metabolic alterations attributable to subclinical disease (68)^6^; thus, inexplicable decreases in cholesterol levels which do not result from lifestyle changes might be an indicator of metabolic dysregulation induced by early malignancy. A recent retrospective study examining abnormal radiotracer uptake in PET-CTs revealed that tumor activity and volume correlated positively with BAT activation in non-cachectic cancer patients (70), indicating that NST—as a presumed mechanism of cachexia—is activated in the early stages of tumor progression, but appears as unintentional weight loss only once the increased energy expenditure can no longer be compensated.

The research findings on the SNS and noradrenergic signaling lead us to infer that a tumor uses the neuroanatomical structures mediating NST, including sympathetic efferents, to render this acute stress mechanism chronic. Moreover, as a mechanism of both tumor-induced changes to bodily homeostasis and tumor-induced immunosuppression, the SNS provides a link between cachexia and cancer biology. Pursuing this line of reasoning implies that the tumor might interfere with the central neural pathways of thermoregulation.

### Central Neural Pathways of Thermoregulation

Under normal conditions, thermal inputs from various body sites are transmitted to central thermoregulatory neuronal circuits located in the preoptic area (POA) of the anterior hypothalamus (43). Thermogenic activation occurs when cold stress activates neurons in the median POA (MnPOA), which in turn decrease the activity of warm-sensitive neurons in the medial POA (MPOA), thereby inhibiting their tonic suppression of sympathetic premotor neurons in the rostral raphe pallidus nucleus.

Which of the thermoregulatory effector mechanisms is activated depends on the duration of cold stress and specific body temperature thresholds. Whereas, NST and tachycardia are rapidly provoked by skin cooling, shivering thermogenesis (ST) is activated only when core body temperature is at stake. This finding has led to the suggestion that the different cold defense mechanisms are mediated by distinct neuron populations (43).

**Hypothesis**: Mediation of cold defense mechanisms by distinct neuron populations provides a rationale for why NST—but not other cold defense mechanisms, such as cutaneous vasoconstriction or piloerection—has been observed to be activated in cancer cachexia. In normal physiology, sympathetically mediated tachycardia is always co-activated as a cardiac thermogenic mechanism along with both NST and ST (43). Assuming that a tumor co-opts the neurocircuits that induce NST in response to cold stress, one might expect to see tachycardia in cancer patients as well. Although the co-occurrence of NST and tachycardia has not been directly studied, an increased resting heart rate has indeed been described in patients with advanced colorectal, pancreatic cancer as well as NSCLC and suggested as an independent predictor of a poor chance of survival (71).

Might the tumor activate hypothalamic neurocircuits, which, by means of sympathetic efferents, trigger BAT activation, WAT browning, and increased lipid mobilization? Although the findings on cancer cachexia inducing low-grade BAT activation that fluctuates to circadian rhythms are still ambiguous, the chronic effects of cachexia on WAT (browning and increased lipolysis) have emerged more clearly. In answer as to how that might be implemented at the neurophysiological level, we refer to what is known about the normal mechanisms involved in thermoregulation and explore these intersections next.

### Histamine Signaling

Histamine signaling plays a role in POA-mediated thermoregulation (72), which depends on the site and receptor type, as shown in rodent models (17). Hyperthermia can be induced by experimentally raising histamine levels in the MPOA and MnPOA (73), which involves H_1_R or H_3_R (74) and H_2_R signaling (75), respectively. Moreover, it has been suggested that H_2_R and H_3_R histamine signaling maintains hyperthermic tone (76, 77). However, histamine signaling also has been implicated in hypothermic effects, although only in rather harsh experimental settings, such as exposure to ionizing radiation (78) or in association with anaphylaxis (79). Notably, hypothermic effects do not involve the POA (17). Histamine signaling in the POA reduces the respiratory quotient, which is an indicator of increased utilization of lipids as an energy source, and increases the expression of uncoupling protein (UCP) in thermogenic tissues, in particular UCP1 in BAT, as well as UCP2 in WAT (73, 80, 81).

**Hypothesis**: WAT browning, and possibly BAT activation, are associated with cancer cachexia. As necessary and sufficient to induce hyperthermia, as well as produce the effects of chronic NST activation, histamine signaling in the POA provides a mechanism by which the tumor might gain control of the thermogenic machinery. Indeed, cancer patients frequently exhibit increased body temperature and night sweats, the latter again pointing to circadian rhythmicity in NST (82).

### Circadian Rhythmicity and Night Sweats

Determining metabolic cycles (83, 84) and body temperature (30), circadian rhythmicity is highly conserved in mammals. In rats^7^, experimentally increasing histamine levels in the MPOA and MnPOA induces significant hyperthermia in their diurnal (inactive) phase, which diminishes in their nocturnal (active) phase (73). This effect likely is mediated by H_1_R activation in the MnPOA, which triggers NST and concomitantly reduces active phase motor activity, thereby relegating maintenance of body temperature toward the NST in histamine-dosed animals vs. toward motor activity in control animals.

**Hypothesis**: If this mechanism were to be active in (human) cachectic cancer patients, it would manifest as a body temperature higher than would otherwise occur with the normal nighttime circadian lowering of temperature. Since the extent of NST activation depends on the degree of histamine receptor activation, it can range from low (e.g., by only compensating the normal drop of body temperature at night, which is prone to remain unnoticed) to the fever described in lung and pancreatic cancer, leukemia/lymphoma and pediatric malignancies, among others (85–88).

Sweating represents a thermoregulatory mechanism which is activated in response to a rise in body temperature. In cancer cachexia, increased histamine signaling might promote thermogenesis while the body's temperature set point remains unchanged. Might cancer patients' night sweats reflect the body's attempt to reduce the differential between the actual—histamine-induced—elevated body temperature and the normal set point temperature, similar to what occurs after anti-pyrogenic drugs are administered to reduce fever? By contrast, inflammation-triggered and prostaglandin E_2_-mediated fever corresponds to the adaptation of the actual body temperature to a set point increased by inflammation. Thus, different neurophysiological mechanisms would underlie cancer cachexia-associated thermogenesis and pyrogenic fever, which is why it is possible to observe simultaneous fever and sweating in cachectic cancer patients.

### The Histaminergic System

The two principal sources of histamine in the brain are mast cells and histaminergic neurons; together they comprise the central histaminergic system (19). Notably, histamine from peripheral sources such as the tumor itself (89, 90) do not affect brain physiology, since histamine cannot pass the blood-brain barrier, but is produced *de novo* from histidine (21). However, the expression of histamine by tumors underlines its dual role in malignancies (91).

Brain mast cells are important mediators of neuroinflammation (92), which has been implicated in cancer cachexia (13, 14). Recent research indicates that activation of brain mast cells induces their degranulation, which in turn activates microglia, and thereby mediates neuroinflammation, and that suppressing mast cells' degranulation prevents neuroinflammation (92). Therefore, the involvement of brain mast cells and thus, histamine signaling, is consistent with neuroinflammation. Moreover, there is evidence for an association between systemic and neuroinflammation (13).

Histaminergic neurons (19–21, 93) are located in the tuberomamillary nucleus (TMN) of the posterior hypothalamus (19–21, 93). They act as pacemakers, autonomously firing at a low rate, and are excitable by various neurotransmitters including noradrenaline, ATP, serotonin, and orexin. Histaminergic neurons are integral to POA-mediated thermogenesis and the regulation of food intake (17). Indeed, stimulating hypothalamic histamine signaling in distinct nuclei of the hypothalamus has been shown to promote NST and anorexia, both major symptoms of cancer cachexia (17, 18).

**Hypothesis**: Systemic inflammation and neuroinflammation may play different roles in cachexia than previously thought. Immunoediting, reflecting the interplay of immune system and tumor, is complex and has been divided into three distinct phases (elimination, equilibrium, and escape), whereby many crucial events occur in the local tumor environment, which in turn affect the immune response at the systemic level (94). Beginning in the early stages of cancer, there are physiological changes in plasma cholesterol levels and resting heart rate that are consistent with NST activation. We hypothesize that these effects occur before systemic inflammation and thus also before neuroinflammation. Physiological effects preceding cancer-associated systemic inflammation (but not necessarily local inflammation) point to an inflammation-independent mechanism as the primary central nervous system (CNS) mechanism in cancer cachexia. Hence, neuroinflammation might exacerbate wasting, rather than being one of its primary causes (see above mentioned discussion on PTHrP).

## Cancer Cachexia-Associated Anorexia

Food intake is regulated at multiple sites in the body, including the cortical areas, hypothalamus, and brainstem. Research on the neurobiological aspects of cancer cachexia has been focused mainly on the hypothalamic melanocortin system (13, 14), which integrates signals about nutritional status and the perception of food (95, 96) and consists of two distinct subpopulations of neurons in the arcuate nucleus (ARC): the agouti-related peptide (AgRP) and pro-opiomelanocortin (POMC)-expressing neurons (95), whose function is to project to and modulate the activity of neurons in the paraventricular nucleus (PVN) by means of the melanocortin 4 receptor. A proteolytic cleavage product of POMC, α-melanocyte-stimulating hormone stimulates, while AgRP inhibits, these neurons; in combination, they determine all aspects of food intake (97). The activity of AgRP and POMC neurons is determined by humoral inputs, including anorexigenic leptin and insulin, orexigenic ghrelin, and satiety-mediating hormones, such as cholecystokinin.

Neuronal histamine signaling is integral to the regulation of food intake (14, 18). The active sites of histamine in this context are the hypothalamic ventromedial nucleus (VMN) and the PVN, but not the lateral hypothalamus, dorsomedial nucleus, or POA. Indeed, the ARC, VMN, and PVN receive histaminergic fibers and markedly express H_1_R (19). H_1_R is expressed in hypothalamic satiety centers and mediates hypothalamic AMP-activated protein kinase (AMPK) activation, while the inhibitory H_3_R inhibits hypothalamic hunger centers (98, 99). The spatial specificity of histaminergic neuron signaling with respect to their biological functions neuroanatomically mirrors the different ways that cancer cachexia manifests, with some patients developing taste or smell distortions, many patients developing anorexia, and some not experiencing these symptoms.

### Increased Sensitivity to Leptin, Decreased Sensitivity to Ghrelin, and the Beneficial Effect of Anamorelin in Cancer Cachexia

**Figure 1:**
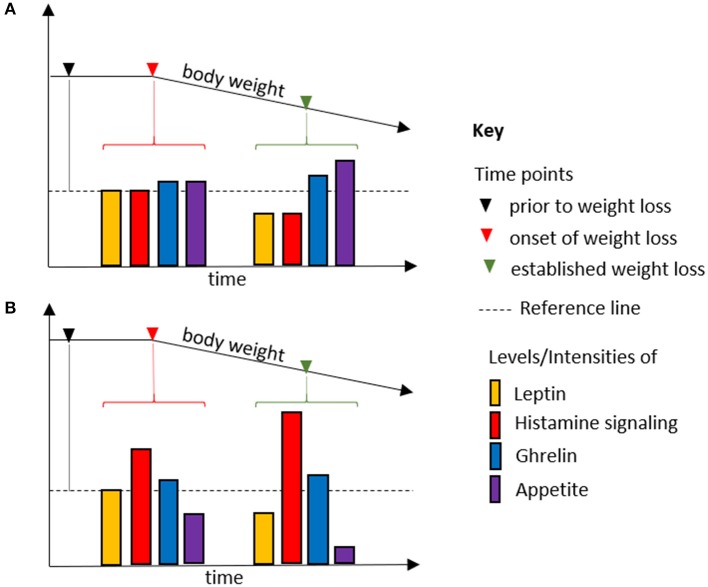
Body weight loss due to fasting **(A)** and in cancer cachexia **(B)**. Histamine signaling is downstream and an integral part of the anorexigenic effect of leptin. As an adiposity sensor, leptin plasma levels, and thus histamine signaling, mirror the course of weight loss during fasting and in cancer cachexia. Analogously, average ghrelin levels are increased in **(A,B)**. However, whereas histamine signaling is determined by leptin levels in fasting, it is uncoupled from it in cancer cachexia. Thus, relative levels of leptin and ghrelin promote appetite in fasting, but not in cancer cachexia. Increased histamine signaling overrules the hormonal regulatory mechanisms in food intake, allowing anorexia to be established as the default state.

One of the functions of neuronal histamine signaling is mediating factors that regulate food intake, such as leptin, orexin, and glucagon-like peptide 1 (19, 100). Histamine signaling being downstream of the anorexigenic hormones leptin and amylin suggests a mechanism of action in cancer-associated anorexia (101–103). We surmise that excessive histamine signaling can overrule humoral signals regulating food intake, thereby inducing and perpetuating an appetite-suppressed state. Plasma leptin correlates with fat mass and cell size and is considered to be an adiposity sensor (104). Cachectic cancer patients present with hypoleptinemia, which, contrary to what one would expect, fails to promote food intake (104). Tonic histamine signaling might account for the apparent increase of sensitivity to anorexigenic factors, above all leptin, associated with cachexia (6) (Figure 1).

Cancer cachexia also has been associated with a decreased sensitivity to orexigenic factors. Since food intake is determined by the balance of orexigenic and anorexigenic factors, a shift toward its tonic suppression would manifest in an apparent loss of sensitivity to orexigenic stimuli. Indeed, plasma levels of ghrelin are increased in cancer patients, but fail to promote food intake, a condition referred to as ghrelin resistance (6). Ghrelin is not directly involved in histamine signaling (105), which might explain why the ghrelin analog anamorelin has proved promising in the treatment of cancer cachexia in advanced clinical studies (106). If ghrelin's orexigenic effect were to require reduced neuronal histamine signaling, this therapeutic approach would not work.

In summary, histamine signaling can induce the main symptoms of cancer cachexia, increased REE and anorexia. In normal physiology, histamine is both a neuromodulator and a neuroinflammation mediator. Considering that inflammation is local rather than systemic in the early stages of cancer and that anti-inflammatory intervention cannot completely abolish wasting once systemic inflammation has been established in advanced cancer progression, our considerations so far support the view that neuronal histaminergic system is involved in the development of cachexia in cancer patients. This mechanism can provide a basic tonus toward wasting, which can be further exacerbated by tumorkines and (systemic and central) inflammation during cancer progression, with this combination of variables contributing to the different manifestations of cachexia in cancer patients. Moreover, the neuronal histaminergic system by itself provides a physiological basis for symptomatic heterogeneity, particularly with regard to relatively minor symptoms.

## Minor Symptoms of Cancer Cachexia

### Diversification of Histaminergic Neurons and Symptomatic Heterogeneity in Cancer Cachexia

Histaminergic neurons are located within the TMN, which is interconnected with almost every part of the brain and the spinal cord. Specifically, the anterior part of the hypothalamus, including the POA, is particularly rich in histaminergic fibers (17). Histaminergic neurons also project to brain areas regulating food intake. Moreover, TMN is innervated by neurons of the brainstem including serotoninergic cell groups, which have already been associated with cachexia-associated anorexia (12, 13, 107). Histaminergic neurons are organized into distinct pathways in terms of origin, terminal projections, and function (108).

The occurrence and severity of minor symptoms vary between individual cancer patients (2). Moreover, the sheer range and heterogeneity of disorders associated with aberrant histaminergic neuron signaling—including hypersomnia, cognitive dysfunction, and Parkinson's disease—suggests the involvement of distinct histaminergic neuronal populations (19, 21). Furthermore, histaminergic neurons can act as stress sensors integrating and responding to a variety of different inputs, including from the PSNS. Recent studies have demonstrated the existence of distinct subpopulations of histaminergic neurons, which specifically target distinct regions of the brain for histamine release (20, 109), and differ in their responsivity to stressors such as restraint, metabolic, hypercapnic, and food deprivation stress, as well as in their sensitivity to neurotransmitters and cannabinoid receptor 1 agonists (110–116). For instance, histaminergic neurons can be divided into two subpopulations with respect to their sensitivity to GABAergic inputs from the ventrolateral POA (VLP) (108), which largely determines sleep and wakefulness (21, 117).

Notably, and concordant with our hypothesis, histaminergic neurons provide the neurobiological prerequisites to induce various clinical manifestations of cancer cachexia.

### Change in the Perception of Taste and Smell

Cancer patients commonly report changes in the perception of taste and smell; the severity of these symptoms correlates with weight loss, reduced food intake, and lower quality of life scores (118, 119). These changes in perception not only are a side effect of chemotherapy and radiotherapy, but also occur in a significant proportion of treatment-naive patients with solid tumors, where they are significantly associated with fatigue and early satiety (120–122). Hypothalamic histamine release is increased by adverse taste stimuli such as saltiness or bitterness, and decreased by stimuli mediating sweetness (123, 124). Normally, taste perception is mediated by the chorda tympani nerve, which is excited by perception of the physical substance (123). It appears that aberrant histamine signaling can invert this mechanism by rendering the default state of food perception to be unpleasant. Indeed, cancer patients complain about a bad taste in the mouth and report taste distortions and occasionally an increased sensitivity to odors (119). There is scant evidence linking smell perception and histamine signaling, but some research findings show that the scent of grapefruit and lavender oil affects autonomic neurotransmission and blood pressure by means of histaminergic neurons (125). It remains to be proven, but would not be unreasonable to presume that enhanced histamine signaling might also influence the perception of smell, not just taste.

### Sleep Disturbances

Cancer patients occasionally experience problems sleeping (2). Neuronal histamine signaling is crucially involved in the regulation of sleep and wakefulness (20). Histaminergic neurons are part of an extensive neuronal network; they include orexinergic neurons, which are located in the perifornical area in the posterior lateral hypothalamus, locally and functionally closely associated with the TMN. Hypothalamic orexins are involved not only in leptin signaling, but also in regulating sleeping and waking (20, 126). The lack of orexinergic neurons induces narcolepsy. Histaminergic neurons are highly active during waking and sluggish or dormant during sleep. Their rate of firing, and thus the extent of histamine release, is regulated by GABAergic neurons of the VLP, which project to the TMN and are complementarily active during slow-wave sleep, the deepest phase of sleep.

The physiological role of histamine as an integral part of orexin signaling points to its possible role in the sleep disturbances of cancer patients and links it to leptin signaling, and thus anorexia and the circadian rhythmicity of NST in cancer cachexia.

## Implications of the Role of the Neuronal Histaminergic System as Tumor Sensor

The physiological function of neuronal histamine signaling and the correlation of tumor activity and BAT activation are consistent with the idea that neuronal histamine signaling could be tumor-triggered, gradually increasing as cancer progresses from the early or subclinical to the advanced stages. The three most significant features of the neuronal histaminergic system which would imply that histaminergic neurons in the TMN might be critically involved in sensing the tumor and thus link tumor growth to cachexia progression are: (1) histaminergic neurons are stress sensors, which (2) differ in their sensitivity to stressors or stress intensities, and (3) provide functional specificity.

**Figure 2:**
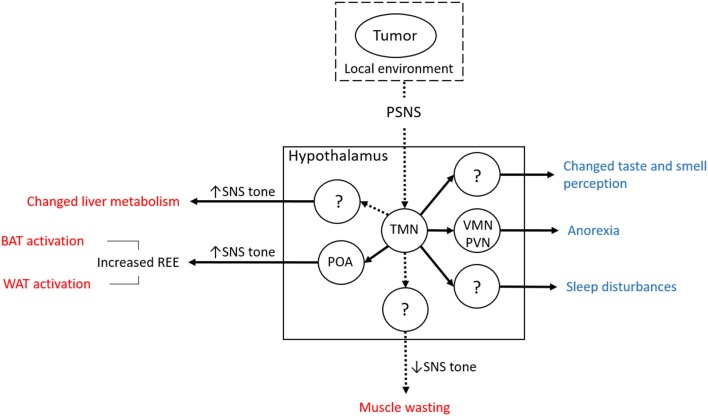
Overview of the hypothesis concerning the involvement of the neuronal part of the histaminergic system in cancer cachexia. Muscle wasting is suggested to involve the loss of SNS tone. The mechanism and the time of onset in the course of cancer cachexia is still not clear, although the switch from increased to reduced neuronal histamine signaling might delineate the late phase (muscle wasting). The notion of histaminergic neurons in the TMN mediating cancer cachexia is consistent with a mechanism involving the sensing of tumor-derived signals (including those reflecting changes in its local tissue environment via the PSNS). Since this mechanism does not rely on systemic inflammation as a trigger of neuroinflammation, it represents an alternative route for tumors to affect systemic metabolism and cause cancer cachexia (BAT, brown adipose tissue; POA, preoptic area; PSNS, parasympathetic nervous system; PVN, paraventricular nucleus; REE, resting energy expenditure; SNS, sympathetic nervous system; TMN, tuberomamillary nucleus; VMN, ventromedial nucleus; WAT, white adipose tissue). Solid lines originating from the TMN indicate that connections have already been shown although in other contexts than cancer cachexia. Dashed lines indicate that while there is no direct evidence, it can be inferred indirectly. Solid lines between circles (hypothalamic nuclei and the periphery, BAT and WAT) indicate that increased SNS tone has been shown to be decisive in the respective physiological context. Central effects of tumor-induced changes of neuronal histamine signaling are shown in blue font, peripheral effects in red.

The neuronal histaminergic system represents an alternative, inflammation-independent mechanism of how tumors can be sensed and how they could induce changes at the systemic level (13) (Figure 2). This mechanism is presumed to be based on tumor-to-brain signaling mediated by the parasympathetic nervous system (PSNS), as has been postulated for serotoninergic signaling (12). The vagus nerve conveys information from visceral regions to the brain (127), including, as we and others suspect, from subclinical tumors (12, 128). An analogous signaling path has been implicated in heart failure, whereby vagal afferent signaling is induced by pressure-sensitive atrial volume receptors in the wall of the right atrium. This signal is then conveyed to the PVN, which responds by increasing sympathetic tone, resulting in heart failure (129).

Histaminergic neurons are responsive to a variety of peripheral signals. For instance, oleoylethanolamide (OEA) is released by enterocytes in response to fat intake and mediates satiety via hypothalamic mechanisms (130, 131). OEA activates sensory fibers of the vagus nerve, which projects to the nucleus tractus solitarii (NTS), where the signal is relayed toward the PVN. Noradrenergic NTS-PVN projections activate the hypothalamic oxytocin system, which mediates the anorectic effect of OEA (132). Moreover, OEA induces expression of c-Fos, a marker for neuronal activation, in a subgroup of TMN neurons, thus betokening the concept of neuronal diversification. Current research supports the idea that OEA might increase histamine levels in the PVN, which in turn stimulates oxytocin release through H_1_R (133).

We posit that the neuronal histaminergic system might be crucially involved in PSNS-mediated tumor sensing and in mediating cancer cachexia, in that it responds to afferent PSNS signals, which in turn complement efferent neuronal mechanisms influencing tumor biology (134). Based on the evidence, the vagus nerve might convey the physical, biochemical, and/or inflammatory traits of the tumor and/or its environment (134) to a subpopulation of histaminergic neurons, which primarily activate NST via the SNS. In the course of cancer progression, more and more functionally distinct histaminergic neurons, specifically those projecting to the PVN and mediating anorexia, become activated, as well as secondary mechanisms involved in modulating cancer cachexia. Following this line of reasoning would suggest that the varying prevalence of cancer cachexia with respect to tumor entities reflects organ-specific differences in parasympathetic innervation (135).

## The Liver and Central Histamine Signaling

Evidence for the involvement of central histamine signaling in the dysregulation of the hepatic metabolism in cancer cachexia is still lacking. Nevertheless, it might be inferred for several reasons:

1. Cancer cachexia is associated with the dysregulation of the hepatic glucose and lipid metabolism (6, 8), which is under the control of the autonomic nervous system, including the SNS under normal conditions (136, 137);
2. Metabolic and humoral signals from the periphery converge with brain-derived inputs in cachexia-relevant brain areas such as the NTS and PVN, where histamine exerts its normal function;
3. Together, BAT, WAT, and the liver constitute a functional unit in chronic NST under normal conditions (65, 138–140).

Point 3 is especially persuasive in support of the histaminergic neuron signaling playing a role in increasing the sympathetic tone in the liver.

## When Sympathetic Tone Breaks Down—Muscle Wasting in Cancer Cachexia

The impact of histamine signaling on muscle physiology and muscle wasting in cancer cachexia has not been studied yet. However, skeletal muscle is the effector organ of ST, which is activated when NST is insufficient to maintain body temperature (43, 141). Both ST and NST mechanisms are triggered by neurons of the POA and involve SNS activation, which provides the link between hypothalamic histamine signaling and noradrenergic signaling at the periphery. Sarcopenic obesity being associated with both reduced neuronal sensitivity to histamine and muscle atrophy underscores the importance of sympathetic tone, i.e., noradrenergic signaling, for skeletal muscle integrity and implicates histamine signaling (142).

The CNS-SNS skeletal muscle axis plays an important role in muscle wasting in cancer cachexia. ACT-ONE, a recent multicenter phase II study of cachectic patients with colorectal cancer and NSCLC, proved the impact of catecholamine-mediated signaling on muscle wasting and loss of lean body mass (143, 144). Under administration of different doses of espindolol^8^, study participants' showed significant increases in body weight and, at the higher dose tested, lean body mass with no effect on fat mass (143).

Espindolol and similar β_2_-adrenoceptor agonists have demonstrated beneficial effects on muscle wasting (145). In an animal model of age-related muscle wasting, espindolol increased muscle mass, and decreased fat mass (146). In another study, espindolol increased grip strength, a measure of skeletal muscle function, in cachectic cancer patients (143). Non-specific beta blockers have been shown to improve the survival rates of ovarian cancer patients (147) and to prevent wasting of skeletal and heart muscles in animal models (148, 149). In sum, both pharmacologic stimulation of β_2_-adrenoceptor and exercise have proven to be beneficial as part of a multimodal approach for the treatment of cancer cachexia (42).

Khan et al. recently described a mechanism which links β_2_-adrenoceptor-mediated signaling to muscle function (150), which underscores the likelihood of its involvement in the muscle wasting experienced by cachectic cancer patients. Their research showed that the integrity of neuromuscular junctions depends on their innervation by sympathetic neurons (151, 152). Indeed, signaling via cAMP/PKA, components of the signaling pathway of noradrenaline, have been found to be important for synapse maintenance (153–157). Sympathetic nerve stimulation has been shown to promote the importation of PPARGC1A, an activator of mitochondrial biogenesis, into myonuclei (158, 159), which underlies the positive effect of muscle training on health promotion and disease mitigation (160, 161).

Increased sympathetic tone mediates cachexia-related alterations of adipose tissue and liver metabolism, whereas decreased sympathetic tone is associated with muscle wasting. Changes in sympathetic tone might accompany distinct stages of cancer cachexia progression, with the first stage characterized by increased sympathetic tone inducing NST, WAT browning, and increased lipolysis and causing adipose tissue depletion; and the second stage characterized by loss of sympathetic tone causing muscle wasting. This would signify (1) that adipose tissue depletion precedes muscle wasting, which has indeed been found in cachectic cancer patients (162, 163), and (2) that histamine signaling, be it of neuronal or mast cell origin, is abolished in the second stage of cancer cachexia, which is supported by research showing that reduced neuronal histamine sensitivity has been implicated in obesity, which in turn is associated with sarcopenia (i.e., obese sarcopenia) (17, 142). The mechanism could be that reduced histamine signaling causes reduced SNS tone in muscles, which could engender the disintegration of neuromuscular junctions, thereby the loss of motor-neuronal inputs, leading to further functional and structural muscle loss.

## Do Histaminergic and Serotoninergic Neurons Team up in Cancer Cachexia?

Serotonin is associated with cancer-associated anorexia (12, 13, 107). Tumor resection indeed brings about the normalization of serotonin signaling with respect to serotonin levels and receptor abundance, while intra-hypothalamic injection of a serotonin antagonist exerts an anti-anorexigenic effect in tumor-bearing rats (13). Histaminergic neurons in the TMN and serotonergic neurons in the raphe nuclei of the brain stem are intensely interconnected (21), with serotonergic neurons directly excited by histamine via H_1_R activation and suppressed via H_2_R (21). In turn, the TMN receives projections from aminergic nuclei, among others, and serotonin excites histaminergic neurons via 5-hydroxytryptamine receptor 2C (21). Serotonin and histamine appear to exert complementary effects on food intake, with serotonin mediating satiety and histamine reducing appetite (13, 17). By establishing a positive feedback loop between aminergic neuron populations, the feature of mutual excitation points to a possible mechanism of dysregulation in cancer cachexia, whereby the activation of histaminergic neurons might trigger the activation of serotoninergic neurons. This sequence of events seems all the more plausible when considering that histaminergic neurons can act as stress sensors, manifesting many clinical symptoms of cancer cachexia, in contrast to serotonin, which primarily affects food intake.

## Caveat: Pharmacologically Targeting the Central Histaminergic System to Treat Cancer Cachexia?

Histamine signaling has been implicated in tumor biology and antihistaminic treatment^9^ is now viewed as an auspicious approach in cancer therapy (14, 164, 165). Still, this promising new treatment must be approached with caution, as pharmacologically interfering with brain histamine signaling may lead to undesirable side effects due to histamine's physiological role as a neuromodulator of a variety of neurotransmitters (19–21, 93). Nonetheless, in a positive development, mirtazapine—an anti-depressant which not only targets noradrenergic and serotonergic signaling but also interferes with histamine signaling—counteracted chemically induced cachexia in mice and, in combination with olanzapine, an antagonist of several neurotransmitter receptors including H_1_R, yielded good anti-nausea results in a phase II clinical trial (166–168). Cyproheptadine, an antagonist of both serotonin and histamine signaling, in combination with megestrol acetate to counteract skeletal muscle wasting, demonstrated an appetite-enhancing effect in pediatric cancer patients (169), but failed to abate progressive weight loss in advanced cancer patients (170–172). In terms of isolating the precise mechanism of action of each individual drug, however, it is not possible to infer the specific role of histamine signaling in this context.

## Discussion

For this work, we scoured publications to gather evidence to support (or disprove) our hypothesis that the neuronal histaminergic system might be involved in cancer cachexia.

Although the data skew positively in support of our hypothesis, we want to anticipate two presumably major objections:

1. It is likely that one or the other line of our argumentation is not commonly accepted. This is certainly true for BAT activation as a mechanism of REE. In this particular case, the controversy on that topic is part of our argumentation and even supports our hypothesis.
2. The issues are not covered appropriately in terms of depth and/or detail and some aspects, especially empirical evidence, are missing. What we aimed to do in this paper is outline the conceptual framework around our hypothesis and its implications, and we hope we have accomplished this in a manner to engage the reader and spark discussion as to the merits of our line of reasoning.

The topic requires at least a high-level understanding of a variety of different disciplines, including oncology, endocrinology, and neurophysiology, which makes it a Herculean task to survey every possibly relevant finding. Fortunately, this was not necessary to draft a useful conceptual framework based on testable assumptions, since the selection of issues was mainly dictated by the line of inquiry. The depth of coverage of each topic, in particular the basic information, was discretionary and reflects the authors' personal choice.

## Conclusion

We would like to conclude by summarizing the cornerstones of our hypothesis. We hypothesize that hypothalamic histaminergic neurons are stimulated early in cancer development via the PSNS sensing tumor traits. Histamine release in the POA primarily activates BAT, which is a highly energy-demanding mechanism. Chronic activation leads to browning of WAT, which further enhances thermogenic potential. In this view, the tumor hijacks the neuronal circuit and parts of the effector system of adaptive thermogenesis. In this context, chronicity refers to intermittent and/or low-grade activation by the SNS. Anorexia, as well as minor symptoms of cachexia, such as taste and smell distortions and sleep disturbances, can be ascribed to aberrant histamine signaling. These functions are exerted by different structures of the brain, which are targeted by distinct histaminergic neuron populations.

As cancer advances, further mechanisms such as systemic inflammation^10^, neuroinflammation, or tumorkines might gain importance and promote cachexia progression. Histaminergic neurons remain active as long as the tumor is present, although their significance for the manifestation of cachexia might lessen. Thus, patients with unresectable cachexia-inducing tumors lose weight even when receiving the optimal multimodal therapy. We hypothesize that in late-stage cachexia, the sympathetic tone in skeletal muscle breaks down, which might be caused by a reduction in histamine signaling or by the interference of other mechanisms in cancer cachexia. Histamine signaling thus might delineate distinct stages of cachexia progression, with the early phase marked by increased histamine signaling, increased sympathetic tone and adipose tissue depletion, and the late phase characterized by reduced histamine signaling, decreased sympathetic tone, and muscle wasting. A possible mechanism of muscle wasting in cancer cachexia mimics that of sarcopenia in obesity, whereby the sympathetic tone in muscle tissue is reduced, leading to the disintegration of neuromuscular junctions. The loss of histamine sensitivity of distinct neurons in obesity might be another candidate for a mechanism behind muscle wasting in cachexia. Pharmacologically targeting neuronal histamine signaling might result in intolerable side effects due to its physiological role as a neuromodulator of a variety of fast-acting neurotransmitters.

Our hypothesis provides a framework for the mechanisms that might be driving cachexia. There is no pharmacological treatment for the histaminergic system currently available, so for now this represents an attempt to better understand this seemingly intractable side effect of cancer. However, if it should prove to be true that energy-demanding mechanisms are activated early in cancer development, it might be possible to exploit the resultant effects, such as a decrease in cholesterol in the absence of substantiating lifestyle changes, for the detection of early malignancy and possible implementation as a screening tool.

## Data Availability Statement

All datasets generated for this study are included in the article/supplementary material.

## Author Contributions

HZ, EZ-T, and MP equally contributed to the conceptualization of the hypothesis presented here as well as its implications for cancer diagnosis. HZ wrote the manuscript, which was discussed with and proof-read by EZ-T and MP.

### Conflict of Interest

The authors declare that the research was conducted in the absence of any commercial or financial relationships that could be construed as a potential conflict of interest.

## Footnotes

^2^There is increasing evidence that histamine signaling in the hypothalamus is involved in the development of obesity (17).

^3^BAT is detected via its uptake of labeled glucose. If this occurs at low levels, BAT will not be detected by ^18^F-FDG-PET-CT.

^4^Either 2 h/day for 6 weeks at 17°C; or up to 6 h/day at 15–16°C for 10 days.

^5^SNS-associated noradrenergic signaling also has been implicated in cancer-associated immunosuppression (67).

^6^And citations therein.

^7^Rats are nocturnal, so their circadian rhythmicity will be the opposite of humans'.

^8^Espindolol is a non-specific beta blocker with pro-anabolic, anti-catabolic, central appetite-stimulating, and thermogenesis-reducing effects via its β_2_-adrenoceptor-stimulating and β_1_-adrenoceptor- and 5-HT1α-receptor-blocking activity (143, 144).

^9^Peripheral anti-histaminic treatment, not CNS anti-histaminic treatment.

^10^Please note: We are not implying that inflammation is not present in the subclinical stage. It may very well be present at a low-grade level. As cancer progresses, it takes on an increasingly prominent role.

## Abbreviations

AgRP: agouti-related peptide
AMP: adenosine monophosphate
AMPK: AMP-activated protein kinase
α-MSH: α-melanocyte-stimulating hormone
ANS: autonomous nervous system
ARC: arcuate nucleus
ATGL: adipose triglyceride lipase
ATP: adenosine triphosphate
BAT: brown adipose tissue
BMI: body mass index
cAMP: cyclic AMP
CCK: cholecystokinin
c-Fos: Fos proto-oncogene
CNS: central nervous system
DMN: dorsomedial nucleus
^18^F-FDG-PET-CT: ^18^F-fluorodeoxyglucose positron emission tomography/computer tomography
FFA: free fatty acids
GABA: gamma-aminobutyric acid
GLP-1: glucagon-like peptide 1
H_1_R, H_2_R, H_3_R: histamine receptor H1, histamine receptor H2, histamine receptor H3
HSL: hormone-sensitive lipase
5-HT_2C_R: 5-hydroxytryptamine receptor 2C
IL-6: Interleukin 6
LH: lateral hypothalamus
MC4R: melanocortin 4 receptor
MnPOA: median POA
MPOA: medial POA
NMJ: neuromuscular junction
NSCLC: non-small-cell lung cancer
NST: non-shivering thermogenesis
NTS: nucleus tractus solitarii
OEA: oleoylethanolamide
PTHrP: parathyroid hormone-related protein
PGE2: prostaglandin E_2_
PKA: protein kinase A
POA: preoptic area
POMC: pro-opiomelanocortin
PSNS: parasympathetic nervous system
PVN: paraventricular nucleus
REE: resting energy expenditure
rRPA: rostral raphe pallidus
ST: shivering thermogenesis
SNS: sympathetic nervous system
TAG: triacylglycerol
TMN: tuberomamillary nucleus
UCP: uncoupling protein
VLP: ventrolateral POA
VMN: ventromedial nucleus
WAT: white adipose tissue
ZAG: zinc-alpha_2_-glycoprotein.
